# To determine correlation between biochemical parameters of nutritional status with disease severity in HCV related liver cirrhosis

**DOI:** 10.12669/pjms.341.14011

**Published:** 2018

**Authors:** Asma Chaudhry, Kaleem Ullah Toori, Javaria Ilyas Shaikh

**Affiliations:** 1 Dr. Asma Chaudhry, MRCP (UK). Department of Medicine, KRL Hospital, Islamabad, Pakistan; 2 Dr. Kaleem Ullah Toori, FRCP (Glasgow). Department of Medicine, KRL Hospital, Islamabad, Pakistan; 3 Dr. Javaria Ilyas Shaikh, MBBS. Department of Medicine, KRL Hospital, Islamabad, Pakistan

**Keywords:** Biochemical parameters, Child-Turcotte-Pugh score, Hepatitis C, Liver cirrhosis, Malnutrition

## Abstract

**Objective:**

To identify correlation between biochemical parameters of nutritional status with disease severity in HCV related liver cirrhosis in patients attending tertiary care hospital.

**Methods:**

Total 259 HCV related liver cirrhosis patients who attended the outpatient department of KRL Hospital, Islamabad from June 2016 to January 2018 were included in this cross-sectional study. HCV status was confirmed with PCR. Cirrhosis was pre-established by ultrasound, while cirrhosis severity was gauged by CTP score. Biochemical parameters for nutrition status included serum albumin, creatinine, cholesterol, LDL, HDL, triglycerides, hemoglobin, ferritin, sodium, potassium, magnesium and calcium. Other demographic and clinical data were also recorded.

**Results:**

The mean age of patients was 58.73 ± 6.04 years with 57.1% being males. The average BMI was 22.72 ± 1.69 kg/m^2^. Majority patients i.e. 123 (47.5%) belonged to CTP-A, 67 (25.9%) were in CTP-B and 69 (26.6%) in CTP-C groups. Significant negative correlations of cirrhosis severity were established with BMI, albumin, creatinine, cholesterol, LDL, TG, HDL, hemoglobin, sodium and magnesium indicative of malnutrition. Analysis of biochemical parameters amongst individual cirrhosis groups revealed significant negative correlation across the same factors in group CTP-C, while CTP-A correlated positively with these parameters. The only significant correlation found in CTP-B was with albumin, HDL, hemoglobin, sodium and magnesium.

**Conclusion:**

Considering limitations of standard ways alone to assess malnutrition in liver cirrhosis, biochemical parameters are valid to aid in diagnosing malnutrition.

## INTRODUCTION

Pakistan has a high prevalence of hepatitis C (HCV), which is one of the key causes of cirrhosis.^1^ HCV related liver disease is a major cause of morbidity and mortality, imposing heavy burden on health care facilities.^2^ Malnutrition is a common concomitant in cirrhosis, being an adverse prognostic factor.^3^ It is commonly missed during monitoring of cirrhosis^4^, hence is a significant problem to be identified. This is especially important in developing countries where the prevalence of malnutrition is quite high.

According to the European Society for Clinical Nutrition and Metabolism (ESPEN) guidelines^5^, subjective global assessment (SGA) and anthropometry are the best ways to evaluate malnutrition in cirrhosis. SGA combines dietary history and clinical examination. However this is difficult in our population as no dietary and weight records are maintained. In addition, usually there is wrong estimation of dietary habits. A Brazilian study^6^ conducted for assessment of malnutrition revealed efficacy of SGA being only 28%. Anthropometry is a quantifiable way to assess malnutrition, however in liver diseases, due to generalized edema, these measurements become less significant. Also there is great variability in the experience of clinicians which can affect outcome of anthropometric measures.^7^ In such cases, a combination of biochemical and clinical factors can be used if SGA and anthropometry are inconclusive.^5^ Various biochemical parameters can be used to assess nutritional status including albumin, creatinine, lipid profile, hemoglobin, ferritin and electrolytes in cirrhosis. Identifying these as indicators of nutritional status may lead to earlier detection of malnutrition. This is essential for timely intervention and improved prognosis of cirrhotic patients. As biochemical parameters are affected by disease process itself, it is important to review how these parameters alter with disease severity.

Port *et al* used several biochemical parameters to assess malnutrition in cirrhosis patients.^8^ Previous Pakistani studies by Ismail et al.^9^ and Naqvi et al.^10^ displayed high prevalence of malnutrition in advancing cirrhosis using SGA and Royal Free Hospital Global assessment scheme respectively. However no study has been carried out in Pakistan to evaluate role of biochemical parameters for assessment of malnutrition. Considering that malnutrition in HCV related cirrhosis is under diagnosed, we planned this study to determine the correlation between biochemical parameters of nutritional status and disease severity in cirrhosis, to help aid early diagnosis of malnutrition.

## METHODS

This cross-sectional study was carried out in HCV related cirrhosis patients being treated in the Medical outpatient department, KRL Hospital Islamabad. The study was conducted from June 2016 to January 2018. The participants were sampled by non-probability technique. According to the literature, the expected prevalence of malnutrition in liver cirrhosis was 85.56%.^10^ Sample size of 259 was estimated with 90% power and 95% confidence level. All stable patients above 18 years of age and of either gender with confirmed HCV related cirrhosis were included. Clinical diagnosis of cirrhosis was documented according to past physician record along with duration of diagnosis. HCV status was established by presence of positive anti-HCV Antibody by ELISA, confirmed subsequently by PCR. Patients who were PCR positive were included in study. Liver cirrhosis had been documented by history, clinical findings, laboratory parameters (complete blood picture, liver function test and coagulation profile) and imaging (ultrasonographic evidence of shrunken liver, coarse echotexture, splenomegaly or ascites).^11^ Cirrhosis severity was scored according to Child-Turcotte-Pugh score (CTP) A to C.^12^ History and examination were carried out to assess ascites, as well as to confirm inclusion and exclusion criteria. Patients with other causes of cirrhosis, current acute illness or chronic medical illnesses like diabetes, chronic kidney disease and congestive cardiac failure were excluded.

Ethical approval was taken from the hospital ethical committee. Written informed consent was taken from all patients. Patients' baseline information like age, sex, duration since diagnosis and BMI were noted using a proforma. Blood samples were taken and the laboratory parameters for biochemical assessment of nutrition with normal ranges were as follows: Serum albumin (3.5-5.5 g/dl), creatinine(0.5-1.5 mg/dl), cholesterol(130-200 mg/dl), Low Density Lipoprotein (LDL) (<150 mg/dl), High Density Lipoprotein (HDL) (30-65 mg/dl), Triglycerides (TG) (35-170mg/dl), Hemoglobin (14-18g/dl (Male), 12-16 g/dl (Female)), Ferritin(Male: 30-400 ng/ml, Female:13-150 ng/ml), sodium(136-149 mEq/L), potassium(3.5-5.0 mEq/L), magnesium(1.6-2.5 mEq/L) and calcium(8.6-10.2 mg/dl). All investigations were carried out according to routine procedure and standard biochemical methods of laboratory at KRL Hospital.

Statistical Package for Social Sciences (SPSS) Version 22.0 was used for data analysis. In descriptive statistics, mean and standard deviation were calculated for continuous variables like age, BMI, duration since diagnosis and biochemical parameters. Frequency and percentages were calculated for categorical variables like gender, cirrhosis severity according to CTP groups and ascites. Pearson's correlation coefficient was used to correlate biochemical parameters and the disease severity according to CTP groups. P-value less than 0.05 was considered significant.

## RESULTS

A total of 259 cirrhosis patients were analyzed. The mean age of the patients was 58.73 ± 6.04 years. Out of 259 patients, 148 (57.1%) were males and 111 (42.9%) females. The average BMI was 22.72 ± 1.69 kg/m^2^. The mean duration since diagnosis was 7.44 ± 3.76 years. There were 123 (47.5%) patients in CTP-A, 67 (25.9%) in CTP-B and 69 (26.6%) in CTP-C. Demographic data are presented in Table-I, while mean values of biochemical parameters for all cirrhosis patients and individual groups per CTP classes are presented in the Table-II.

**Table-I:** Basic characteristics of sample data (n=190).

	Cirrhosis Patients
Age (in years) – Mean ± SD	58.73 ± 6.04
*Gender- n(%)*	
Male	148 (57.1)
Female	111 (42.9)
BMI (kg/m2)- Mean± SD	22.72 ± 1.69
*CTP – n(%)*	
A	123 (47.5)
B	67 (25.9)
C	69 (26.6)
Ascites- n(%)	142 (54.8)
Duration since diagnosis (years)- Mean ± SD	7.44 ± 3.76

**Table-II:** Mean values of biochemical parameters of all cirrhosis patients and individual cirrhosis groups as per CTP Class.

Biochemical parameters	All Cirrhosis patients Mean ± SD	CTP-A Mean ± SD	CTP-B Mean ± SD	CTP-C Mean ± SD
Albumin (g/dl)	3.34 ± 0.76	4.01 ± 0.33	3.17 ± 0.18	2.33 ± 0.29
Creatinine (mg/dl)	1.09 ± 0.33	1.20 ± 0.24	1.09 ± 0.31	0.90 ± 0.40
Cholesterol (mg/dl)	155.78 ± 38.97	170.86 ± 45.71	148.02 ± 24.22	136.43 ± 24.37
LDL (mg/dl)	139.99 ± 23.46	143.74 ± 22.51	138.49 ± 25.87	134.75 ± 21.77
Triglycerides (mg/dl)	137.25 ± 26.63	144.26 ± 27.81	131.92 ± 23.46	129.91 ± 24.46
HDL (mg/dl)	51.62 ± 9.72	55.26 ± 8.46	48.14 ± 8.93	48.50 ± 10.33
Hemoglobin (g/dl)	11.21 ± 1.98	12.22 ± 1.98	10.39 ± 1.19	10.21 ± 1.72
Ferritin (ng/ml)	138.58 ± 34.27	136.39 ± 32.94	138.98 ± 35.75	142.11 ± 35.33
Sodium (mEq/L)	134.90 ± 4.03	137.78 ± 2.80	133.53 ± 2.50	131.10 ± 3.15
Potassium (mEq/L)	3.96 ± 0.32	3.96 ± 0.28	3.90 ± 0.29	4.02 ± 0.40
Magnesium (mEq/L)	1.73 ± 0.20	1.87 ± 0.16	1.67 ± 0.16	1.53 ± 0.11
Calcium (mg/dl)	9.42 ± 0.36	9.39 ± 0.36	9.44 ± 0.33	9.44 ± 0.40

Correlation of biochemical parameters of malnutrition with all cirrhosis patients and individual groups is shown in Table-III. Significant negative correlations were established with albumin, creatinine, cholesterol, LDL, TG, HDL, hemoglobin, sodium and magnesium. BMI also was also found to inversely correlate with cirrhosis severity. Longer duration of diagnosis was linked with increasing cirrhosis severity (r=0.75, p=0.00). When individual groups' correlations were reviewed, significant negative correlation was found across the same factors only in group CTP-C. On the other hand, CTP-A correlated positively with the same factors. The only significant correlation found in CTP-B was with albumin, HDL, hemoglobin, sodium and magnesium.

**Table-III:** Correlation of BMI and biochemical parameters across all cirrhosis patients and individual cirrhosis groups.

	All cirrhosis patients r (p)	CTP-A r (p)	CTP-B r (p)	CTP-C r (p)
BMI	-0.239* (0.00)	0.225* (0.00)	- 0.056 (0.36)	-0.198* (0.001)
Albumin	-0.925* (0.00)	0.835* (0.00)	-0.138* (0.02)	-0.806* (0.00)
Creatinine	-0.359*(0.00)	0.303*(0.00)	-0.006(0.92)	-0.336*(0.00)
Cholesterol	-0.379*(0.00)	0.369*(0.00)	-0.118 (0.05)	-0.300*(0.00)
LDL	-0.163*(0.009)	0.153*(0.01)	-0.038 (0.54)	-0.135*(0.03)
Triglycerides	-0.238*(0.00)	0.251*(0.00)	-0.118 (0.05)	-0.166*(0.007)
HDL	-0.316*(0.00)	0.357*(0.00)	-0.211*(0.001)	-0.193*(0.002)
Hemoglobin	-0.451*(0.00)	0.484*(0.00)	-0.245*(0.00)	-0.304*(0.00)
Ferritin	0.069 (0.26)	-0.061 (0.32)	0.007 (0.91)	0.062 (0.31)
Sodium	-0.709*(0.00)	0.681*(0.00)	-0.201*(0.001)	-0.570*(0.00)
Potassium	0.061 (0.32)	-0.005 (0.93)	-0.104 (0.09)	0.109 (0.08)
Magnesium	-0.689* (0.00)	0.645*(0.00)	-0.157*(0.01)	-0.573*(0.00)
Calcium	0.062 (0.32)	-0.07 (0.26)	0.04 (0.52)	0.039 (0.53)

* p<0.05 was considered significant

## DISCUSSION

Malnutrition in liver cirrhosis frequently leads to increased mortality risk.^13^ The frequency of malnutrition in cirrhosis is variable, affecting 50%-90% of patients.^3^,^10^ Nutritional assessment is difficult in cirrhosis as parameters for evaluation of malnutrition are affected by cirrhosis itself. A previous Pakistani study assessed malnutrition by SGA^9^; instead we used biochemical parameters to assess malnutrition. As in other studies^9^,^14^ the predominant gender was male in our study population. In our study, most patients were in CTP-A group, similar to a Brazilian study^8^, the most likely explanation being an outpatient study with stable cirrhotic patients. The same study by Port et al.^8^, evaluated the association between biochemical parameters of nutritional assessment and CTP groups in HCV related cirrhosis, finding significant reductions in cholesterol, albumin and creatinine. We found albumin, creatinine, cholesterol, LDL, TG, HDL, hemoglobin, sodium and magnesium to reduce significantly with increasing cirrhosis severity. Biochemical parameters are affected by both liver cirrhosis and malnutrition. However, on studying individual groups, CTP-A showed a rise in values of these parameters while CTP-C showed decline in same parameters. This variability suggests that liver cirrhosis alone does not impact biochemical parameters, but rather malnutrition has an independent effect on these parameters as well. Ismail et al.^9^ pointed that nutritional status worsens as disease severity increases and our results showed similarity. As malnutrition has worst impact in advancing cirrhosis in CTP-C patients^10^, the parameters are most significantly affected in this subgroup. Hence these parameters can be used to aid the diagnosis of malnutrition in cirrhotic patients. The reliability of BMI to assess nutrition status is variable in cirrhosis due to fluid retention and falsely high BMI. BMI can be a reliable indicator of malnutrition with different cut-off points depending on ascites.^6^ In our study however, it was seen that BMI reduced with increasing severity of cirrhosis. This needs to be looked into with further studies studying BMI changes with severity of ascites and cirrhosis simultaneously.

Reduced levels of cholesterol, HDL, LDL and TG are a feature of liver cirrhosis as reported previously by an Iranian study.^15^ This Iranian study also stated that while cholesterol, HDL and LDL significantly reduced with worsening severity of cirrhosis, TG levels did not. Another Indian study found that except TG, all other lipid variables were low.^16^ Our study reported significant reductions in cholesterol, HDL, LDL and TG with increasing severity of cirrhosis. Significant reductions in TG levels in our study can be due to the reason that we only studied HCV related cirrhotic patients and HCV is known to cause reduction in all lipid levels even in the absence of cirrhosis.^17^ However malnutrition is a contributory factor as well. Lipids can thus be used to assess progression of liver disease indicative of development of malnutrition.

In this study, we found hemoglobin to decrease significantly in advancing cirrhosis as studied by Port et al.^8^ Regarding ferritin metabolism, a Turkish study^18^ found ferritin reduction with increasing severity however our results differed. This was surprising as ferritin is a marker of inflammation and is expected to rise in chronic inflammatory conditions like cirrhosis.^19^ However as we did not have iron or transferrin levels, it is difficult to comment why this disparity exists.

As regards metabolism of minerals, we found significant drop in magnesium and sodium with increasing cirrhosis severity. The abnormality in sodium was expected as it's a well-documented fact that cirrhosis causes hyponatremia.^20^ We also found magnesium to reduce with liver cirrhosis, similar to a study conducted by Nangliya^21^ revealing that liver dysfunction affects the metabolism of trace elements like magnesium. A reduced magnesium level as with all other minerals is an indicator of malnutrition. As our study suggested, magnesium decreases significantly in both CTP-B and CTP-C. This may be an early indicator of malnutrition emerging in CTP-B group. Finally, creatinine and albumin, as in previous literature^8^,^22^ were low in our study as well, and alone are less relevant indicators of malnutrition.

This is the first Pakistani study that highlights the importance of biochemical markers like cholesterol, LDL, HDL, TG and magnesium as a gauge of nutritional status as opposed to previous studies which used SGA alone to assess malnutrition.^9^,^10^ Although affected by liver disease itself, there were differences amongst the cirrhosis severity groups. This implies that these markers can be used to aid timely diagnosis of malnutrition. This has new implications as SGA may be difficult to perform on every patient in clinics and has performer variabilities as discussed. Biochemical parameters instead are performed frequently in patients and can highlight earlier detection of malnutrition. Considering the high prevalence of malnutrition, this is an important step leading to reduction of morbidity.

### Limitation of the study

Lack of comparative group as it was a cross-sectional study. Also, biochemical parameters were not compared with the usual malnutrition assessment tools. Further studies are needed where biochemical parameters along with SGA or anthropometric measures are carried out simultaneously.

## CONCLUSION

Although the biochemical markers can't be used alone to diagnose malnutrition, they can be of additional value in patients where other assessments e.g. SGA or anthropometry can't be carried out due to limitations. This would maximize the benefits of earlier detection of malnutrition, allowing intervention as promptly as possible to reduce morbidity of cirrhotic patients.

### Authors Contribution

**AC** conceived, designed and did statistical analysis, data collection & editing of manuscript.

**AC & JIS** designed the questionnaire, data collection and manuscript writing and editing.

**KT** contributed to study design, data interpretation, review and final approval of manuscript.
